# Patients’ conceptions of opting out of group-based education for type 2 diabetes in Swedish primary care – a phenomenographic interview study

**DOI:** 10.1080/17482631.2026.2731703

**Published:** 2026-09-11

**Authors:** Susanne Andersson, Mia Berglund, Anna Kjellsdotter, Annelie Bäckström, Ylva Persson, Sofia Dalemo

**Affiliations:** a Department of Health Sciences, University West, Trollhättan, Sweden; b Research, Education, Development & Innovation, Primary Health Care, Region Västra Götaland, Sweden; c School of Health Sciences, University of Skövde, Skövde, Sweden; d Department of Research, Development, Education and Innovation, Skaraborg Hospital, Skövde, Sweden; e Primary Health Care Centre Närhälsan Ågårdsskogen, Lidköping, Sweden; f Primary Health Care Centre Närhälsan Guldvingen, Lidköping, Sweden; g General Practice, Institute of Medicine, Sahlgrenska Academy, University of Gothenburg, Gothenburg, Sweden

**Keywords:** Diabetes mellitus type 2, education, primary healthcare, phenomenography, self-management

## Abstract

**Purpose:**

Type 2 diabetes affects everyday life, requiring patients to learn to live with the illness. Although group-based education can improve clinical outcomes and support self-care, it remains underutilised in Sweden. Only about a fifth of healthcare centres offer group-based education, and many patients opt out of participation. Understanding the various reasons patients opt out is crucial for designing group-based education that meets individual needs and promotes long-term disease management. This study aims to describe variations in the conceptions of patients with type 2 diabetes who opted out of group-based education.

**Methods:**

A phenomenographic approach was applied based on 18 individual interviews with patients in primary care who opted out of group-based education for type 2 diabetes.

**Results:**

Three conceptions emerged regarding group-based education for type 2 diabetes, reflecting patients’ varied views and understanding. Some patients saw no need for group-based education and preferred individual learning. Others recognised the benefits and barriers of group-based education, such as inflexible schedules. Others appreciated the reflective and supportive environment, shared experiences, and greater self-insight.

**Conclusions:**

Patients who declined group-based education held varied conceptions, from preferring individual counselling to recognising benefits. Understanding these variations supports more personalised education and better long-term self-management. These insights may inform clinical and assistive practices by promoting more flexible and person-centered approaches to diabetes education within primary care.

## Introduction

Having a long-term illness such as type 2 diabetes (T2D) affects everyday life and necessitates learning to live with the illness. In Sweden, approximately 5% of the population has T2D (Swedish National Diabetes Register, 2025), and it is estimated that 783 million adults will have the disease worldwide by 2045 (Chen et al., 2011; Sun et al., 2022). In addition to personal burden, T2D carries an increased risk of serious complications and premature death (Patsoukaki et al., 2025) and is associated with high health expenditures (Sun et al., 2022).

Persons with T2D usually receive care at primary healthcare centres, with individual counselling being the most common patient education method (Bergh et al., 2014). Planned educational activities, individually or in a group, aim to increase health levels, knowledge, and self-care skills, which are needed to achieve disease control and treatment (The National Board of Health & Welfare, 2018). Group-based education, often led by a diabetes nurse, is a valuable arena for reflecting together in a group of people with T2D (Kjellsdotter et al., 2020). Dietitians, physiotherapists, and physicians may sometimes take part. The groups are intended for patients who have recently received a diabetes diagnosis and typically include 6–8 participants. Most group-based education comprises about three sessions, each lasting roughly 2 hours. A key aim is to encourage reflection and change experiences within the group (Kjellsdotter et al., 2020).

There is an important difference between receiving a diagnosis and integrating the illness into one’s life (Hörnsten et al., 2011), and this can be considered a learning process (Berglund, 2011; Johansson et al., 2016). Persons with long-term illness need to integrate their illness into daily life, an understanding that increases the ability to self-care (Johansson et al., 2016). A person-centred approach that considers the needs, values, and opportunities for self-care is important and emphasised by the American Diabetes Association, the European Association for the Study of Diabetes, and the National Institute for Health and Care Excellence (McGuire et al., 2016; van Vugt et al., 2020; Young-Hyman et al., 2016).

Person‑centred care also plays a central role. As Ekman et al. (2011) describe, person‑centred approaches emphasise partnership, shared decision‑making, and recognition of the patient’s lived experience. In group education, this translates into flexible discussions, individualised settings, and respect for participants’ diverse backgrounds and needs.

Improved understanding achieved through person-centred group-based education has been linked to long-term improvements in HbA1c among individuals with T2D (Jutterström et al., 2016). Furthermore, a meta-analysis revealed that group-based education is more effective than traditional individual counselling in terms of outcomes such as HbA1c, fasting blood glucose, weight, and triglycerides (Odgers-Jewell et al., 2017). Group-based educational programmes also provide the opportunity to reflect on and share experiences with others who have T2D (Kjellsdotter et al., 2020).

Diabetes self-management education is a cornerstone of clinical and assistive practice. Contemporary guidance highlights the importance of multidisciplinary and person-centred approaches in diabetes care (Rosenfeld et al., 2025). However, engagement in education and self-care support is influenced by multiple factors, including diabetes-related distress, health literacy, and patient–provider communication, which are associated with self-care behaviours (Misra et al., 2022; Nam & Yoon, 2024). Against this background, understanding how patients who opt out of group-based education conceive its relevance may inform the development of more flexible and person-centred diabetes education in primary care and support healthcare professionals in tailoring education to individual patients’ needs.

Although group-based education is recommended (The National Board of Health & Welfare, 2015) and often a prerequisite for effective self-care, several diabetes clinics do not offer group-based education. A large nationwide cross‑sectional study found that only around 20% of patients with T2D are offered group‑based education (Husdal et al., 2018). A report on group‑based patient education for T2D in Swedish primary care further revealed that only 16% of healthcare centres provide such programmes, often due to limitations such as insufficient time, limited interest, and lack of pedagogical competence (Swedish Association of Diabetes Nurses, 2024).

Although group-based education is effective, diabetes nurses note that many patients offered group-based education choose not to participate (Husdal et al., 2018; The National Board of Health & Welfare, 2015). Research is lacking on why patients opt out of group-based education. Thus, it is necessary to understand why some patients choose not to participate. This study aims to describe variations in the conceptions of patients who opt out of participating in group-based education in connection with a T2D diagnosis.

## Materials and methods

### Design

A descriptive qualitative design with a phenomenographic approach was used to explore variations in patients’ conceptions of opting out of group-based education for T2D. Phenomenography is a qualitative research method that aims to examine variations in phenomena rather than describe them. To describe how people perceive, understand, or conceptualise a phenomenon, it is therefore necessary to reflect on qualitative differences (Marton, 1986). The primary purpose is to describe the variation in conceptions within a group. Although based on individual experiences, the results of the phenomenographic study are described on a group level (Marton & Booth, 1997). The study adhered to the guidelines of the Consolidated Criteria for Reporting Qualitative Research (COREQ) checklist (Data S1) (Tong et al., 2007).

This approach is particularly relevant for describing variations in the conceptions of a phenomenon—in this case, opting out of participating in group-based education for T2D (Sjöström & Dahlgren, 2002). Phenomenography is commonly used in nursing research to explore such variations (Dalemo et al., 2024). The study examined the collective variation in patients’ conceptions to identify different aspects of the conceptions of declining group-based education at the time of diagnosis. Understanding these variations may provide valuable insights into how patients who opt out of group-based education perceive their decisions (Marton & Booth, 1997).

### Settings and participants

Nurses at two publicly run primary healthcare centres in rural areas of two southwestern Swedish cities, both offering group-based education, identified potential participants. Each centre served between 11,000 and 15,000 registered patients. Both health centres were in smaller towns with between 30,000 and 40,000 inhabitants.

The inclusion criteria for the study were as follows:

- Received a T2D diagnosis within the past year before the interview
- Aged 18 years or older
- Had been offered but opted out of participating in group-based education at the healthcare centre
- Possessed sufficient Swedish language proficiency to participate in an interview

While 33 persons were invited to participate in the study, only 20 individual interviews were conducted, as 13 people declined the invitation. Two of the interviews were later excluded due to insufficient language proficiency. See Table I for details of the interviewees’ characteristics. The sample size was deemed sufficient to explore diverse perspectives.

**Table I.** Sociodemographic characteristics of interviewed patients with recently diagnosed type 2 diabetes mellitus who declined participation in group-based education.

Number of patients	18
*Male/Female (count)*	8/10
*Age (year)*	
Range	40–74
Median	59
*Marital status (count)*	
Married, cohabiting	13
Partner living apart	1
Single	4
*Education (count)*	
Elementary school	5
High school	10
College	3
*Duration of interview (A4 pages)*	
Range	4–36
Mean	9

#### Data collection

Due to COVID-19, the interviews were conducted over a long period from 2019 to 2025. Nine face-to-face interviews were conducted before the COVID-19 pandemic, and afterwards, the remaining nine were done via telephone.

Three of the authors, who are experienced nurses (SA, AB, and YP), conducted the interviews, which were numbered in the order they were done. The participants were between 40 and 74 years old (Table I). Two patients were excluded because they needed a language interpreter. Slightly more women than men participated, and the patients had diverse marital statuses and educational levels.

The semi-structured interviews were recorded digitally and transcribed verbatim. An interview guide was used; see the appendix, and some of the questions included were ‘Can you tell us about how you gained the necessary knowledge to manage your everyday life with diabetes?’ and ‘Can you tell me more about your choice not to participate in the group lessons you were offered?’. Furthermore, open-ended and follow-up questions, such as ‘Please, tell me more’ and ‘What is your experience?’, were used to clarify the intended meanings behind the patients’ answers.

**Table II.** The seven steps performed during the analysis, according to Sjöström and Dahlgren (2002).

1. Familiarisation	All transcribed data − 166 pages in total (A4 double line spacing) − were read several times by two of the authors (SA, SD) to become familiar with the data and obtain a sense of the whole.
2. Compilation	The interviews from all respondents regarding their conceptions of group-based education with persons having T2DM who declined participation. Significant statements were analysed in line with the aim of the study by SA and SD. In total, 187 significant units were identified.
3. Condensation	The different statements were grouped and summarised by identifying what they had in common, resulting in representative descriptions of the dimensions.
4. Grouping	Similar representative statements were grouped into the same conception.
5. Comparison	The groups were compared to find similarities and differences in their conceptions.
6. Naming	The conceptions that emerged were discussed and named to express their essentials.
7. Contrastive comparison	The descriptive categories that emerged were compared in terms of similarities and differences. Finally, three conceptions and five dimensions that present the outcome space were found (SA, SD) and discussed among the authors.

#### Analysis

Data analysis (Table II) was conducted according to the phenomenographic method described by Sjöström and Dahlgren (2002). Initially, the data analysis was performed by two authors (SA and SD). During further analysis, all the authors reflected on their preconceptions from earlier experiences of working as professionals, and discussions were held to achieve a common understanding. The sample size was deemed sufficient to explore diverse perspectives, as qualitative research does not aim for statistical representativeness but for depth and richness of data.

#### Ethical considerations

The study adhered to the Declaration of Helsinki (World Medical Association Declaration of Helsinki, 2013), and approval was obtained from the Regional Ethical Review Board [*N* 442-15]. All participants gave oral informed consent, which was recorded, to participate in the study. The participants were informed that they could withdraw from participation without any consequences for their future treatment. It was also emphasised that participation was voluntary and that confidentiality would be maintained.

## Results

Three qualitatively different conceptions regarding group-based education were identified: ‘sees no need for group-based education’, ‘sees both opportunities and obstacles with group-based education’, and ‘sees that group-based education can increase insights and self-awareness’. The first conception reflects a lack of perceived need for group-based education, a preference for individual information, and discomfort in group settings. The second conception reflects ambivalence towards group-based education and difficulties with inflexible schedules. The last conception conveys that group-based education is seen as both educational and supportive, offering valuable experience sharing and fostering self-insight.

The following sections provide a detailed overview of the three conceptions, accompanied by representative quotes from the interviews (Table III).

**Table III.** Outcome space of the analysis.

		Categories of descriptions				
		Sees no need for group-based education	Sees both opportunities and obstacles with group-based education		Sees that group-based education can increase insight and self-awareness	
**Dimensions**	To participate in a group	**Prefers individual education** *‘Group—no thanks. I would rather sit and talk about my diabetes one-on-one’.* *(Interview 4)*	**Hesitations about the group** *‘I didn’t know how big the group was and, therefore, didn’t dare sign up’.* *(Interview 14)*		**The group contributes with different skills** *‘Yes. And that is what is the strength of a group—everyone is in the same situation… to get tips and ideas about what others have done or can do. The diabetes nurse can come in with their knowledge, and the dietitian probably, too. But that is the strength of the group’.* *(Interview 15)*	
	To prioritise the effort	**Does not prioritise the group** ‘*I was offered a—ehhh—she (the nurse) gave me a note about something or other—I neglected, and I put it away (laughter)’.* *(Interview 3)*	**Other commitments are prioritised** *‘Then I was supposed to take this course then, which I said yes to. But then it turned out that it ended up on Wednesdays and Thursdays, and on that day, I go to pick up my grandchild’*. *(Interview 2)*		**Know from experience that groups can be educational** *‘Yes, if I am asked about group education again, I will say yes. I declined because my husband had a stroke and I had to stand in for him... drive her to training and the hospital.... I thought it was just good when I was with my husband in his group education in connection with his stroke’.* *(Interview 6)*	
	To feel safe	**The disease is not perceived as threatening** *‘My wife is well read (laughter). She works as a care assistant in the municipality, so she has a good grasp of it all’.* (Interview 4)	**Have enough support** *‘No, I think it’s started well … the tests once a year. I can call if I feel something is not right. I just called, and I'll come right away’.* (Interview 2)		**Support for integrating the disease into your life** *‘I haven’t thought much about it, but I think that you discuss with the others in the group how they experience their diabetes and that you recognise how others feel about trying to live with the disease. You can’t run away from it’.* (Interview 1)	
	To share experiences	**Not comfortable in a group** *‘Maybe it would have been fun to be with… but at the same time not. I don’t feel like a group person, but I probably just want to be by myself. Big, strong—I can handle myself’.* (Interview 9)	**Do not have any experiences to share** *’It would definitely be good to have group education for me. However, I feel like I don't have any experiences to share, as it is now. I'll take the day as it comes…’.* (Interview 5)		**Increased learning in groups** *’I don't really know what to say about it—thoughts—well it's probably good to be involved—because even though—as I know a bit about diabetes. It can be difficult to see yourself as diabetic—easier with others. Good to meet others who actually struggle with the same concerns as someone else when it comes to diet, exercise, and everyday life’.* (Interview 13)	
	A need for knowledge	**Get knowledge in other ways** *‘I was offered group education, but what the hell—why? I think it is enough what you read in papers and online; there’s always something’.* (Interview 4)	**Know some things, but want to know more** *‘I want to know what I’m doing wrong and what I’m doing right. I've never been much for cookies and sweets... I need to learn more’.* (Interview 10)		**Knowledge through the sharing of experiences** *‘A group with both relatives and those affected... and it gave a lot. So, I think that's important—sharing experiences with others and how they experience it. If they have any problems, I think that's important’.* (Interview 6)	

### Conceptions

This section details the three conceptions identified through the analysis.

### Sees no need for group-based education

Patients who opted out of group-based education expressed that they saw no need for it. They preferred individual counselling and felt that they already had sufficient knowledge through other sources, such as newspapers and the internet. Group contexts were experienced as uncomfortable and irrelevant, as the disease is not considered threatening or serious. One can live a long, healthy life despite the disease. Several patients mentioned that they had many commitments and that group meetings were not a priority. There is a clear preference for dealing with the disease on their own, with support from their own experiences and individual counselling rather than joint discussions in a group.

### Sees both opportunities and obstacles with group-based education

Patients saw both opportunities and obstacles with group-based education, where uncertainty about the group’s size and composition created hesitation. Other commitments in everyday life were often prioritised, making it difficult to participate despite interest. At the same time, some patients felt that they already had sufficient support from the primary healthcare centre and, therefore, did not see an urgent need for group meetings. However, there was a desire to learn more, and group-based education was considered potentially valuable for one’s learning.

### Sees that group-based education can increase insight and self-awareness

Patients considered group-based education a valuable support for increased insight and self-awareness, where the exchange of experiences and joint reflection contributed to learning. The group was experienced as a place where different skills meet, strengthening the understanding of the disease. Previous experiences of group activities had been positive and were considered educational, even for relatives. The group’s support helped to integrate the disease into everyday life and created recognition in others’ experiences.

### Dimensions of the conceptions

The three conceptions were further analysed based on five dimensions, representing central aspects of how patients experience and understand the phenomenon. The following dimensions were analysed: ‘to participate in a group’, ‘to prioritise the effort’, ‘to feel safe’, ‘to share experiences’, and ‘a need for knowledge’. An overview of the iterative analysis process that emerged is presented in the following subsections.

### To participate in a group

There was variation in the breadth of patients’ attitudes towards participating in group-based education. The attitudes ranged from a clear refusal to participate, often due to a preference for individual counselling and discomfort in group settings (sees no need for group-based education), to a more positive attitude towards groups, but with hesitation due to uncertainty about the group and its size (sees both opportunities and obstacles with group-based education). At the more advanced end of the spectrum, patients recognised the potential benefits of group-based education. They highlighted the value of shared experiences and possibilities to reflect individually and together with others as important for learning and support (sees that group-based education can increase insight and self-awareness).

### To prioritise the effort

There were significant differences in how patients prioritised participating in group-based education. Some participants did not prioritise group-based education at all because of other commitments, such as work, family, and leisure (sees no need for group-based education). Others had originally intended to participate in group-based education, but other more important commitments, especially family and work, got in the way (sees both opportunities and obstacles with group-based education). In the last group, patients saw opportunities with group-based education that they, based on previous positive experiences, perceived as educational and valuable. They had a desire to participate in group-based education (sees that group-based education can increase insight and self-awareness).

### To feel safe

The study revealed that patients with T2D perceived themselves as safe when the disease was not considered threatening, especially if they had relatives who had lived long lives with the disease (sees no need for group-based education). Others perceived that they had sufficient knowledge to manage and had good support from the healthcare centre, with access to testing and the possibility of quickly receiving help. This contributed to security despite the disease and reduced the need for group-based education (sees both opportunities and obstacles with group-based education). Some perceived that group discussions in a safe environment, where experiences are shared with others in similar situations, could provide support in accepting and living with the disease (sees that group-based education can increase insight and self-awareness).

### To share experiences

There are significant differences in how patients are willing to share their experiences. Some patients felt uncomfortable in a group setting and preferred to manage their disease on their own (sees no need for group-based education). Some had insight that group-based education could be educational, even if they could not consider contributing to the group by sharing their experiences (sees both opportunities and obstacles with group-based education). Others saw opportunities to share experiences with others in similar situations to increase the understanding of living with an illness and learning about the disease (sees that group-based education can increase insight and self-awareness).

### A need for knowledge

The study revealed variations in how patients sought knowledge about T2D. Some considered information from the media and internet sufficient and, therefore, saw no need for further group-based education (sees no need for group-based education). Other patients perceived a need to understand more about T2D and obtain practical tips, for example, about food (sees both opportunities and obstacles with group-based education). Finally, some patients saw group-based education and sharing experiences with others as a valuable way to deepen their understanding of T2D and gain new insights (sees that group-based education can increase insight and self-awareness).

## Discussion

The first category of description was that patients perceived no need to participate in group-based education. Many had previously identified themselves as healthy persons, so receiving a diagnosis and joining a group with others with T2D represented a significant psychological shift (Beverly et al., 2012). For some, the disease was not perceived as threatening, and group education was, therefore, considered unnecessary (Dalemo et al., 2024), as one can live a long and good life with the disease. Even with additional time to process their diagnosis, many patients in this group remained uninterested in participating in group-based education.

Additionally, some patients newly diagnosed with T2D expressed a preference for individual counselling over group-based formats. They felt uncomfortable in a group setting and indicated a desire to receive knowledge through alternative methods. These patients would probably have been equally averse to participating in any other type of group within healthcare, not just diabetes (Coleman et al., 2024). For these patients, individual counselling appears to be a more suitable alternative, where knowledge can be conveyed in a way that better matches their personal preferences and needs.

The second category of description is that patients perceive both opportunities and obstacles with group-based education. Many of the patients in this group could, with a little adaptation, such as flexible meeting dates or work hours, consider participating in group-based education. Some of them probably needed a longer time to realise that they live with a long-term illness (Kjellsdotter et al., 2020) and imagine participating in group-based education with other people who have recently been affected by the same disease.

Some patients newly diagnosed with T2D felt uncertain about group-based education because they did not know how many people were participating in the group and who the others were. It could be that they considered the disease shameful because it is associated with a sedentary lifestyle and being overweight. They may struggle to recognise their own experience of living with a long‑term illness (Berglund, 2014) and prefer not to participate in group‑based education alongside neighbours or other familiar individuals. This phenomenon is more relevant in smaller towns, where people are more likely to know each other; in larger cities, it is easier to remain anonymous.

The third and final category of description was that group-based education offers an opportunity for reflection and a means to deepen insight and self-awareness. This group described several positive aspects of participating in group-based education. They would participate in group-based education if they were again offered the opportunity to participate at a time that suited them. Several expressed surprise that they were not offered the opportunity to participate in group-based education several more times after they opted out of the first opportunity. However, it could be difficult for healthcare professionals to offer group-based education to patients who have previously refused it. It could be that the healthcare staff who deliver group-based education feel that they lack competence and, therefore, prefer not to provide it (Bergh et al., 2014).

Research shows that group-based education is perceived as beneficial (Stenberg et al., 2016). The learning process itself is complex, and the responsibility lies on both the learner and the educator (Muijsenberg et al., 2023). Since planning and implementing group-based education requires pedagogical competence, time, and structural support, healthcare professionals should be encouraged to offer group-based education (Bergh et al., 2014; Swedish Association of Diabetes Nurses, 2024).

For some patients, accepting group-based education may symbolise acknowledging that they have been affected by the disease—an important step in the learning process and a way to take control and actively steer one’s life with T2D (Kjellsdotter et al., 2020). There is a meaningful distinction between receiving a diagnosis and integrating the illness into one’s life (Swedish National Diabetes Register, 2025). Living with a long-term condition such as T2D affects everyday life and creates a need to learn how to coexist with the illness. This process can be understood as a journey of learning and adaptation (Hörnsten et al., 2011). From a clinical and assistive practice perspective, these findings highlight the need for more flexible and person-centred approaches to diabetes education in primary care, where healthcare professionals actively tailor educational strategies and invitations to match patients’ varying needs, readiness, and preferences for participation.

Phenomenography aims to capture the different ways individuals make sense of a particular phenomenon. This study highlights the diverse understandings expressed by patients with T2D who opted out of participation in group‑based education. It is essential to emphasise that the study does not seek to identify variations in individual experiences; rather, it examines the collective variation reflected in participants’ individual accounts.

Some patients saw no need for group-based education and preferred individual counselling. Other patients expressed ambivalence towards group-based education or recognised its reflective and supportive value. Understanding these variations is essential for tailoring educational approaches that align with individual preferences, promote engagement, and support effective long-term self-management in primary care settings (Stenberg et al., 2016).

### Strengths and limitations

The chosen qualitative method—interviews combined with a phenomenographic approach—was well-suited for capturing the collective variations in experiences among patients newly diagnosed with T2D regarding their conceptions of not participating in group-based education. The aim of phenomenography is not to describe objective reality (Sjöström & Dahlgren, 2002) but to understand a phenomenon by identifying and structuring the different ways it is perceived (Åkerlind, 2023). Trustworthiness was ensured throughout the analysis by the authors discussing the collective variations to achieve consensus among themselves (Lincoln & Guba, 1985).

Three of the authors participated as interviewers, which could be considered a potential limitation. However, dependability was supported using a structured interview guide containing consistent questions and techniques. Furthermore, all the interviewers had several years of experience meeting patients in a primary care context, which contributed to the quality and consistency of the data collection process. Even though the study and interviews were conducted over several years due to the COVID-19 pandemic, we observed no differences in conceptions over time.

However, the question remains as to whether the study included a representative sample of patients who opted out of group-based education. Those who opt out of group-based education would probably also opt out of participating in a study. Thus, conducting the interviews individually instead of in a group was likely a significant advantage for this group, as they had chosen not to participate in group-based education.

In phenomenographic studies, it is common for no new conceptions to emerge after approximately 10 to 12 interviews (Larsson & Holmström, 2017), a notion supported by the findings of this study. The data collected were rich and diverse, providing a solid foundation for identifying meaningful variations in patients’ conceptions. The conceptions did not differ over time, even though the study and interviews were conducted over several years due to the COVID-19 pandemic.

To enhance the study’s credibility, the analysis was conducted collaboratively by the authors, who established categories of description through analytical discussions. To support the credibility of the findings, illustrative quotations were presented in Table III, allowing for the validation of the relevance and authenticity of the participants’ experiences.

It could be a disadvantage that both health centres in this study were in smaller cities. Patients in larger cities may hold different conceptions, as they may not have previously known each other within the group. In smaller municipalities, the participants could be even more negative towards group education because they are familiar with most of the people living in the village. Patients may feel uncertain about the group composition that is not known to them in advance.

When considering the results’ transferability to similar settings, it is important to acknowledge the possibility that other experiences may emerge, as individuals vary in how they perceive situations (Åkerlind, 1981). This result will vary over time and is therefore not a stable truth. The results of this study are transferable to Swedish primary care, but probably also to Nordic primary care. Semi-structured interviews were selected as the data collection method and were conducted within one year of T2D diagnosis. This timing is considered a strength, as it allowed respondents sufficient time for reflection without the risk of forgetting key aspects of their experiences.

As the incidence of T2D continues to increase (Sun et al., 2022), individual counselling in connection with a T2D diagnosis will likely become less common due to limited resources. Consequently, a greater number of patients should be invited to participate in group-based education than is currently the case. Some patients value having clear, practical details, such as the group’s composition, the schedule and location, who will lead the sessions, and what is expected of participants, including their involvement in discussions (Odgers-Jewell et al., 2017; Stenberg et al., 2016). Providing this information could make them more likely to agree to take part in group‑based education. This may influence patients' choices not to participate in group education. This could be addressed by allowing patients to choose which group they want to be included in, so they can make their own choice about group affiliation.

Patients who opt out of the initial invitation should be offered group-based education again, as the first opportunity simply may not have aligned with their circumstances. Healthcare providers should feel confident in extending multiple invitations, even when patients have previously opted out. If healthcare providers perceive group-based education as time‑consuming and feel uncertain about their pedagogical competence (Bergh et al., 2014; Swedish Association of Diabetes Nurses, 2024), they may be more inclined to avoid offering new invitations to group sessions to patients who have previously opted out of group-based education.

The group that ‘sees no need for group-based education’ will probably remain difficult to engage despite considerable effort (Stenberg et al., 2016; Swedish Association of Diabetes Nurses, 2024). Those who ‘see both opportunities and obstacles with group-based education’ will probably accept an invitation when the timing feels right (Kjellsdotter et al., 2020). The group that ‘sees that group-based education can increase insight and self-awareness’ balances between possibilities and obstacles to participation (Berglund, 2014; Johansson et al., 2016). These groups can vary over time, but the pattern aligns with findings from another study (Andersson et al., 2008).

One potential approach is to allow patients to book themselves online for a session at a time that suits them and when they feel ready to participate (Kjellsdotter et al., 2020; Odgers-Jewell et al., 2017). Another approach could be to offer group-based education across a larger geographical area (Husdal et al., 2018). This would ensure that the nurses charged with educational responsibilities possess appropriate pedagogical competence and motivation (Bergh et al., 2014).

## Conclusion

This study has identified and described various perspectives of patients who opted out of participating in group-based education in connection with a T2D diagnosis. While some saw no need for group education and preferred individual counselling, other patients expressed ambivalence, and some recognised its reflective and supportive value. As patient experiences vary significantly, it is not feasible to approach everyone the same way. Understanding such variations is essential for tailoring educational approaches that align with personal preferences, foster engagement, and support effective long-term self-management in primary care settings.

## Supplementary Material

Supplementary Material Interview guide opting out.docx

Supplementary Material COREQ 32 Patients’ conceptions of opting out 2.docx

## Data Availability

The data that support the findings of this study are openly available in Zenodo at https://doi.org/10.5281/zenodo.18443093.
