# Supplementary material for: Patients’ conceptions of opting out of group-based education for type 2 diabetes in Swedish primary care – a phenomenographic interview study
Source: Int J Qual Stud Health Well-being. 2026 Sep 11;21(1):2731703. doi: 10.1080/17482631.2026.2731703 (PMC13573521; doi:10.1080/17482631.2026.2731703)
Supplement: Supplementary file 1 [file ZQHW_A_2731703_SM7269.docx]

**Consolidated criteria for reporting qualitative studies (COREQ): 32-item checklist**

| **No** | **Item** | **Guide questions/description** |
| --- | --- | --- |
| **Domain 1: Research team**  **and reflexivity** |  |  |
| Personal Characteristics |  |  |
| 1. | Interviewer/facilitator | Which author/s conducted the interview or focus group?  Answer: Three of the authors were involved in the interviewing. |
| 2. | Credentials | What were the researcher's credentials? *E.g., PhD, MD*  Answer: The researcher's credentials were PhD (2), MD (2), MSc (2). |
| 3. | Occupation | What was their occupation at the time of the study?  Answer: 1 physician, 5 specially trained nurses. |
| 4. | Gender | Was the researcher male or female?  Answer: Six females and no males. |
| 5. | Experience and training | What experience or training did the researcher have?  Answer: All researchers had long experience in meeting patients in clinical practice. Four of the researchers also have long experience in research, three of qualitative methods. |
| Relationship with participants |  |  |
| 6. | Relationship established | Was a relationship established prior to study commencement?  Answer: No. |
| 7. | Participant knowledge of the interviewer | What did the participants know about the researcher? e*.g., personal goals, reasons for doing the research*  Answer: The informants were asked to participate by their regular contact at the healthcare centre. In connection with this, they were informed about the purpose of the study, who would conduct the interviews, and why. They were told that those who completed the study worked in healthcare. |
| 8. | Interviewer characteristics | What characteristics were reported about the interviewer/facilitator? e.g., *Bias, assumptions, reasons, and interests in the research topic*  Answer: We have avoided any personal relationship with the informants. Before the study, we discussed our shared pre-understanding as a group. One assumption is that if you opt out, you are not interested in receiving a new invitation to group education. |
| **Domain 2: study design** |  |  |
| Theoretical framework |  |  |
| 9. | Methodological orientation and Theory | What methodological orientation was stated to underpin the study? e.g., grounded theory, discourse analysis, ethnography, phenomenology, content analysis  Answer: A phenomenographic approach was applied to the analysis. |
| Participant selection |  |  |
| 10. | Sampling | How were participants selected? e.g. purposive, convenience, consecutive, snowball  Answer: The inclusion criteria for participants were receiving a T2D diagnosis within the past year before the interview. Aged 18 years or older. Had been offered but opted out of participating in group-based education at the healthcare centre. Possessed sufficient Swedish language proficiency to participate in an interview |
| 11. | Method of approach | How were participants approached? e.g., face-to-face, telephone, mail, email  Answer: Intended participants were informed of the purpose of the participation by their regular health care professional, that participation was voluntary, and that all data would be treated confidentially and stored without access for unauthorised persons. Before the interviews, oral informed consent was obtained from the participants interviewed face to face or by telephone. All interviews were audially recorded and transcribed verbatim. |
| 12. | Sample size | How many participants were in the study?  Answer: A total of 20 individual interviews were conducted, with two interviews later being excluded due to insufficient language proficiency. |
| 13. | Non-participation | How many people refused to participate or dropped out? Reasons?  Answer: 33 persons were invited to participate in the study; 13 persons declined the invitation. Two of the interviews were later excluded due to insufficient language proficiency. |
| Setting |  |  |
| 14. | Setting of data collection | Where was the data collected? e.g. home, clinic, workplace  Answer: All of the face-to-face interviews were conducted at their health care centre at their own choice. The other patients were interviewed by telephone. |
| 15. | Presence of non-participants | Was anyone else present besides the participants and researchers?  Answer: No. |
| 16. | Description of sample | What are the important characteristics of the sample? e.g., demographic data, date  Answer: Age, sex, marital status, level of education, and duration of interviews. |
| **Data collection** |  |  |
| 17. | Interview guide | Were questions, prompts, guides provided by the authors? Was it pilot tested?  Answer: A semi-structured interview guide was used with open-ended and follow-up questions. |
| 18. | Repeat interviews | Were repeat interviews carried out? If yes, how many?  Answer: No. |
| 19. | Audio/visual recording | Did the research use audio or visual recording to collect the data?  Answer: The interviews were audio-recorded and transcribed verbatim. |
| 20. | Field notes | Were field notes made during and/or after the interview or focus group?  Answer: No. |
| 21. | Duration | What was the duration of the interviews or focus group?  Answer: The transcribed interviews varied and were between four and 36 A4 pages. |
| 22. | Data saturation | Was data saturation discussed?  Answer: No, but we considered the data from the study very rich, and the aim of the study was answered. |
| 23. | Transcripts returned | Were transcripts returned to participants for comment and/or correction?  Answer. No |
| **Domain 3: analysis and findings**z |  |  |
| Data analysis |  |  |
| 24. | Number of data coders | How many data coders coded the data?  Answer: Two of the six authors were principal investigators, but all authors read and discussed the results. |
| 25. | Description of the coding tree | Did authors provide a description of the coding tree?  Answer: Not applicable. |
| 26. | Derivation of themes | Were themes identified in advance or derived from the data?  Answer: Conceptions and dimensions emerged from the data. |
| 27. | Software | What software, if applicable, was used to manage the data?  Answer: Excel |
| 28. | Participant checking | Did participants provide feedback on the findings?  Answer: No |
| Reporting |  |  |
| 29. | Quotations presented | Were participant quotations presented to illustrate the themes / findings? Was each quotation identified? e.g. participant number  Answer: The interviews were numbered 1–18, and each quotation in Table 3 is identified by the corresponding interview number. |
| 30. | Data and findings consistent | Was there consistency between the data presented and the findings?  Answer: Yes, there was consistency between the presented data and the findings. |
| 31. | Clarity of major themes | Were major themes clearly presented in the findings?  Answer: Conceptions and dimensions were derived from the data and clearly presented. |
| 32. | Clarity of minor themes | Is there a description of diverse cases or discussion of minor themes?  Answer: No. |
