# Supplementary material for: Patients’ conceptions of opting out of group-based education for type 2 diabetes in Swedish primary care – a phenomenographic interview study
Source: Int J Qual Stud Health Well-being. 2026 Sep 11;21(1):2731703. doi: 10.1080/17482631.2026.2731703 (PMC13573521; doi:10.1080/17482631.2026.2731703)
Supplement: Supplementary file 2 [file ZQHW_A_2731703_SM7270.docx]

**Interview guide for patients’ conceptions of opting out of group-based education for type 2 diabetes in Swedish primary care**

**Gender:**
Male / Female

**Marital status:**
Married/cohabiting / Single

**Age:**

**Educational level:**

- Compulsory school / Folk high school
- Upper secondary school
- College / University

**Living with diabetes**

- How long have you known that you have diabetes?
- What are your thoughts about having been diagnosed with diabetes? (Cause: shame/stigma?)

**Learning**

- Can you tell me about your experiences of learning about diabetes?
- How do you feel about learning together with others in a group compared to learning individually?

**Group-based education**

- Can you tell me about how you gained the necessary knowledge to manage your everyday life with diabetes?
- Do you feel that you have sufficient knowledge to manage your daily life with diabetes? Please, tell me more.
- Please, tell me more about your choice not to participate in the group-based education you were offered?
- Can you tell me about your thoughts regarding your decision to opt out?
  *(e.g., the significance of your own and others’ experiences of group education; your thoughts and feelings about participating in a group with others who have diabetes)*

**Closing**

- Is there anything you would like to add that we have not asked about?

**Follow up questions**

Please, tell me more? How did you feel?

Can you give me some examples?
